# Evaluation of occipitocervical neutral position using lateral radiographs

**DOI:** 10.1186/s13018-014-0087-2

**Published:** 2014-10-05

**Authors:** Jiangwei Tan, Guangjun Liao, Shaoxian Liu

**Affiliations:** Department of Orthopedic Surgery, Yantai Affiliated Hospital of Binzhou Medical University, Yantai, 264000 People’s Republic of China; Department of Orthopedic Surgery, Yantaishan Hospital, No. 91 Jiefang Road, Zhifu district, Yantai, 264000 People’s Republic of China

**Keywords:** Fusion, Mandible cervical distance, Neutral, Occipitocervical, Radiograph

## Abstract

**Background:**

Intraoperative assessment of neutral occipitocervical balance during a fusion procedure is challenging. We designed this study to introduce a more comprehensive method of evaluating the occipitocervical neutral position using lateral radiographs.

**Methods:**

One hundred neutral lateral cervical spine radiographs interpreted as normal were studied. Cervical spine radiographs were performed using a standard technique. The occipitocervical angle, the occipitocervical distance, and the mandible cervical distance were measured by different observers.

**Results:**

A difference analysis was performed between males and females. The mean mandible cervical distances were 11.0 and 11.2 mm in males and females, respectively. The mean occipitocervical distances were 22.0 mm (male) and 19.6 mm (female), and the occipitocervical angles were 47.2° (male) and 45.5° (female). The occipitocervical distance revealed significant differences between males and females (*p* <0.01). However, there were no significant differences between sexes for the occipitocervical angle or the mandible cervical distance (*p* >0.01).

**Conclusions:**

This study offers reference values for the occipitocervical angle and occipitocervical distance for the estimation of the occipitocervical neutral position. The introduction of the mandible cervical distance may make the evaluation more direct and more comprehensive during surgery because of its sensitivity to changes in head position.

## Background

Occipitocervical fusion is indicated for occipitocervical instability caused by trauma [1], bone tumor [2], congenital deformity [3-5], and rheumatoid arthritis [6,7]. It is of great importance to fuse the head in a neutral position during the operation.

If the head is immobilized in a nonfunctional position during occipitocervical fusion, compensatory curvature may occur in the subaxial segments, which will eventually develop into spinal disorders. Toyama et al. studied 12 children who underwent C1-C2 posterior fusions [8]. The authors found that postoperative cervical malalignment (kyphosis or swan-neck deformity) occurred in four patients. In all four patients, new bone formation and an increase in the body/canal ratio (BCR) at the apex of kyphosis were observed. This phenomenon was not observed in eight patients with normal alignment. Matsunaga et al. studied the association between any changes in the alignment of the cervical vertebrae and the development of subaxial subluxation during follow-up periods [9]. Twelve (86%) patients experienced subaxial subluxation after surgery; the authors emphasized the position of the fixed occipital bone and axis during occipitoaxial fusion procedures. Recently, Yoshida reported a case of an upper airway obstruction immediately after an O-C2 fusion in a flexed position [10]. After revision surgery in which the fusion angle was changed, the upper airway obstruction disappeared. Therefore, for surgeons, accurately defining the occipitocervical neutral position before performing occipitocervical fusion is of great importance.

However, reports in the literature that provide a definition of the neutral occipitocervical position are rare. Modern technologies, such as magnetic resonance imaging (MRI) and three-dimensional computed tomography (CT), only complicate the definition, and such technologies are not routinely used during surgery. Radiographs seem to be a last resort for this purpose in terms of practicability. Phillips et al. proposed using the occipitocervical angle (OCA) and the occipitocervical distance (OCD) measurements on lateral radiographs to objectively define the occipitocervical neutral position [11]. However, because OCA and OCD measurements need to be very precise, surgeons still have difficulty in neutral position assessment during surgery when radiographs are not available.

In this study, we introduce the mandible cervical distance (MCD) measurement, which makes the evaluation quick and practical to perform. At the same time, we measured the OCA and the OCD in a larger sample and obtained normal value ranges, which will help to define the occipitocervical neutral position more comprehensively. This study is expected to provide reference values for the definition of the occipitocervical neutral position.

## Methods

We included in this study 100 neutral lateral cervical spine radiographs interpreted by two radiologists and three orthopedic surgeons as normal (i.e., no fracture or dislocation, no deformity, no severe osteophyte formation, no destruction of the vertebrae, no spondylosis) from a radiographic database at Yantaishan Hospital, China. The study population consisted of 50 males and 50 females with an average age of 45.6 years (ranging from 15 to 60 years). X-ray technology was used according to a routine protocol. The subjects were asked to look straight ahead with their mouths closed naturally during projection. The X-ray tube was centered on C_5_, and the radiographs were taken from a distance of 1.8 m. Approval for this study was obtained from the Ethics Committee of the Yantaishan Hospital.

**Figure 1:**
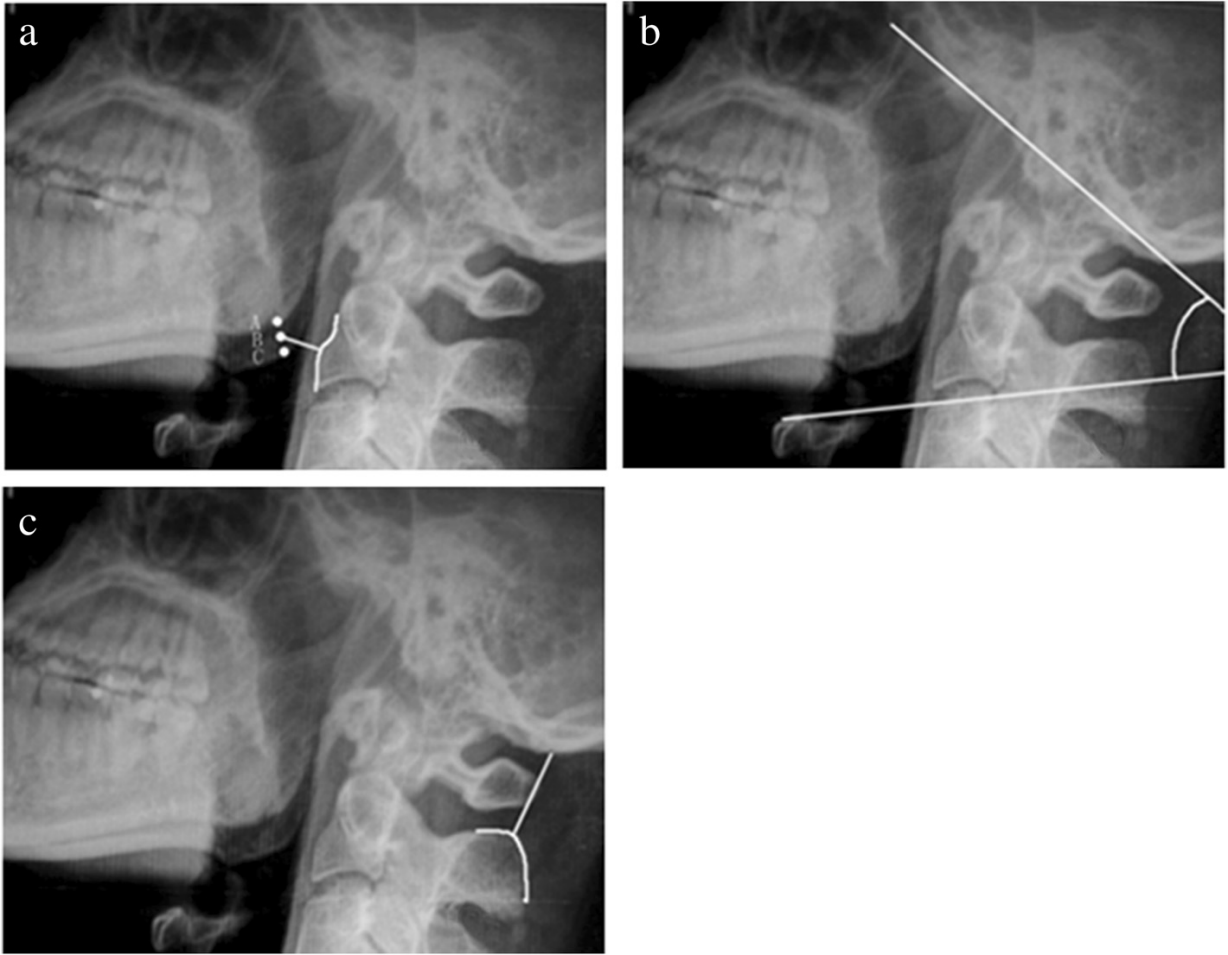
**Illustrations of the MCD, OCA, and OCD on one lateral radiograph.** The MCD is the shortest distance (BD) from the midpoint (point B) of the two apices of the mandible angles (points A and C) to the curve of the anterior border from C_2_ to C_3_ **(a)**. The OCA is the intersection of McRae’s line and a line drawn parallel to the superior endplate of C_3_ **(b)**. The OCD was the shortest distance from the superior aspect of the C_2_ spinous process to the occipital protuberance **(c)**.

The MCD, the OCA, and the OCD were measured on the lateral radiographs. The MCD is the shortest distance (BD) from the midpoint (point B) between the two mandibular angles (points A and C) to the anterior border of C_2_ or C_3_ (Figure 1a). The OCA was defined by the junction of McRae’s line (a line intersecting the basion and the opisthion) and a line drawn parallel to the superior endplate of C_3_ (Figure 1b). The OCD was obtained by measuring the shortest distance from the most superior aspect of the C_2_ spinous process to the occipital protuberance (Figure 1c).

The three parameters were independently measured by the first three authors three times at an interval of 1 month. Mean values were obtained for the final analysis.

Interobserver and intraobserver agreements were assessed using ANOVA. A difference analysis was performed between the men and women using Student’s *t*-test. The confidence interval was set at *p* <0.01. The data were expressed as the mean ± standard deviation (SD).

## Results

### Inter- and intraobserver agreement

There were no significant differences in the MCD, OCD, or OCA among different measurements by the same observer or among different observers (*p* >0.01; Table 1).

**Table 1:** **Inter- and intraobserver agreement for the MCD, OCD, and OCA**

	**MCD**		**OCD**		**OCA**	
	**Intraobserver**	**Interobserver**	**Intraobserver**	**Interobserver**	**Intraobserver**	**Interobserver**
*F* values	0.007	0.001	0.340	0.267	0.050	0.024
*p* values	0.993	0.999	0.712	0.766	0.951	0.976

### Measurements of OCA, OCD, and MCD

OCA, OCD, and MCD measurements were analyzed, and the results are summarized in Table 2. The mean MCDs were 11.0 and 11.2 mm in males and females, respectively. The mean OCDs were 22.0 mm (male) and 19.6 mm (female), and the OCAs were 47.2° (male) and 45.5° (female). No significant differences in OCA and MCD were observed between the genders (*p* >0.01), but a significant difference in the OCD was observed (*p* <0.01).

**Table 2:** **Final results of the measurements of the MCD, OCD, and OCA**

	**Male (mean ± SD)**	**Female (mean ± SD)**	***p*** **value**
MCD (mm)	11.0 ± 2.1	11.2 ± 1.9	0.578
OCD (mm)	22.0 ± 3.4	19.6 ± 4.9	0.005
OCA (°)	47.2 ± 5.0	45.5 ± 4.2	0.077

## Discussion

In this study, we have determined reference values for estimating the occipitocervical neutral position using three key measurements. A comprehensive evaluation using all three parameters should provide an accurate estimation of the neutral position.

Conceptually, the occipitocervical neutral position is the functional and balanced position of the head on the cervical spine. A person feels most comfortable in this position. Radiologically, occipitocervical neutral is defined as the position in which the subject looks straight ahead during a standard lateral cervical radiograph; in this position, the mandible should not overlap C_2_ or C_3_ [12]. All these criteria are subjective, and they are not practicable for patients with injuries or during an operation. Harris et al. and Uno et al. studied the relationship between the occiput and the axis in normal and pathologic states to identify occipitovertebral dissociation [13,14], but they did not establish a measurement to define the occipitocervical neutral position.

The motion characteristics of the occipitocervical segment are obviously different from those of other cervical parts because of the occipitocervical segment’s specific anatomic features. The three-dimensional movements of the occipitoatlantal junction and the atlantoaxial junction meet the requirements for fine movements of the head; at the same time, these movements make it challenging to define the occipitocervical neutral position. Wills et al. used an individualized occipitocervical angle to evaluate the change in lordosis at the occipitocervical junction in patients treated with posterior occipitocervical arthrodesis [15]. This angle can only be used for comparisons in the same person because it is involved in the entry point of the occipital wire and the anteriorinferior corner of the lowest fused vertebral body, which varies in patients. The O-C_2_ angle, created by McGregor’s line and the inferior surface of the axis, has been commonly used in the literature [10,11]. This method can be used to evaluate the outcome of surgical treatment. However, the O-C_2_ angle is quite sharp, and it is not convenient to obtain precise measurements during surgery. The OCA and the OCD proposed by Phillips in 1999 were of clinical significance [8]; however, the OCA is still not easy to measure during surgery, and the OCD varies greatly in different people.

In this paper, we introduced the MCD measurement to make the evaluation process quicker. The MCD is more sensitive than the OCD because the distance from the occipitoatlantal junction (the axis of nodding) to the angle of the mandible is longer than the distance between the occipitoatlantal junction and the occipital protuberance. That is to say, the motion of the head affects the MCD much more than it affects the OCD. We believe that the MCD is more reliable than the OCD because morphologic variations of the C2 spinous process are more common than variations of the mandibular angle. Because the normal value of the MCD ranges from approximately 9 to 13 mm on the basis of our measurements and was approximately half the sagittal length of the C3 vertebral body, it was convenient, in clinical practice, to roughly judge the position by eye based on the ratio of the MCD/sagittal length of the C3 body, which made hardcopy films unnecessary during surgery. For these reasons, we used the MCD as a quick screening method for judging the occipitocervical neutral position in the operating theater.

**Figure 2:**
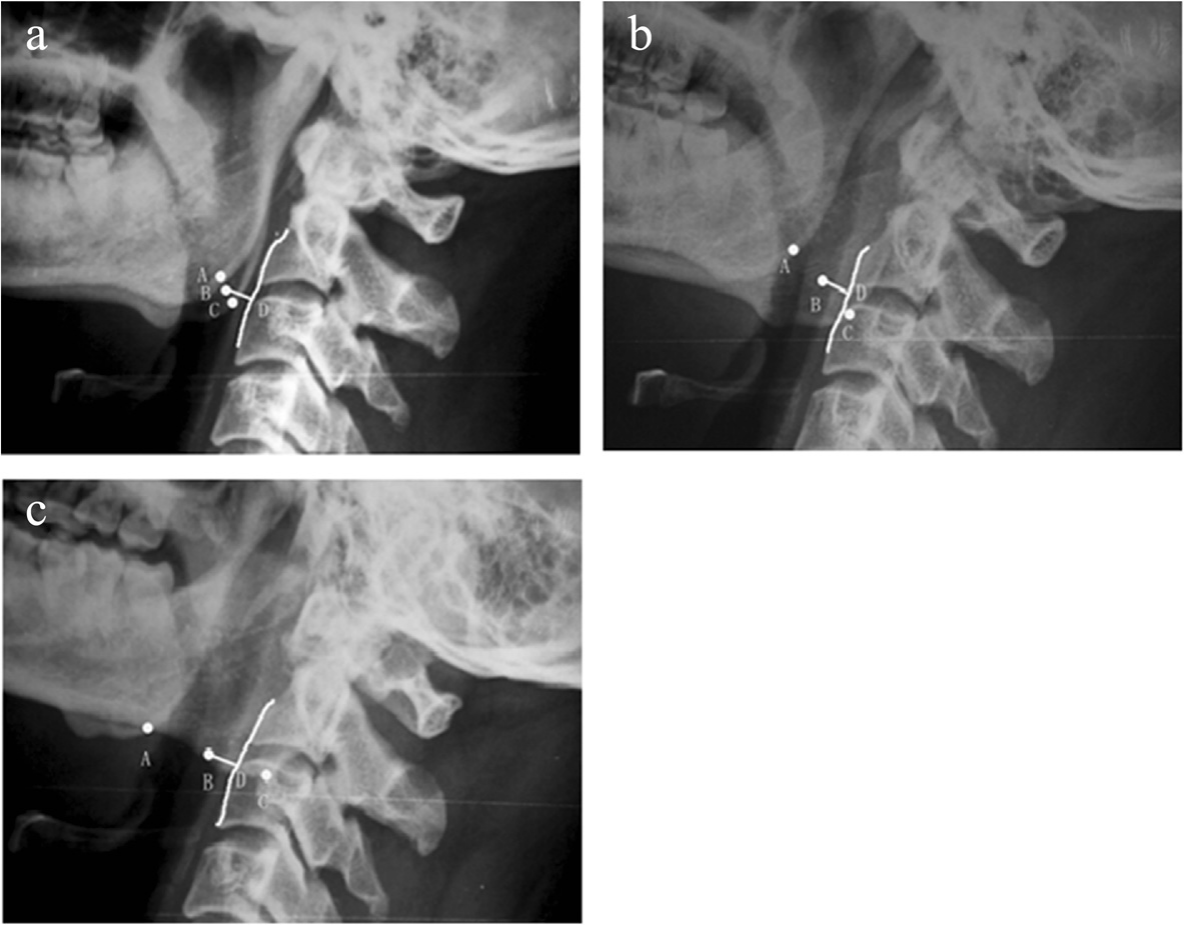
**The MCDs were less affected when the head was slightly rotated.** The figure shows the head in neutral position **(a)**. The head turned right **(b)**, and the head turned left **(c)**.

In our study, we emphasize that the head rotation should be corrected, and the tube should be vertical to the sagittal plane of the head during radiography. On a standard lateral radiograph in neutral rotation, the two mandibular angles are separated from each other by less than 1 cm. However, slight rotation of the head, which is common in clinical practice, can significantly affect the positions of the mandibular angles. We select the midpoint between the two mandibular angles to eliminate this variation (Figure 2a, b).

The OCD revealed significant differences between males and females, whereas the OCA and the MCD did not. Although variations of the C_2_ spinous process may affect the OCD value, we found that variations exist in both males and females and have a tendency to balance. The differences in atlas heights between males and females should be taken into consideration to explain the different OCDs. A conclusion can be made only when the atlas heights in a large sample are measured and statistical comparisons are made. In addition, the tube-film distance in our study was 1.8 m, in contrast to 2.0 m in Phillips’ study. Although the OCD result reported by our group is similar to that found by Phillips’ group, they are not comparable because of the different tube-film distances.

The OCA, OCD, and MCD values correlate with each other during the motion of the occipitocervical junction. For example, in flexion of the head, the OCA and the MCD decrease, while the OCD increases. Although each parameter can define the neutral position to some extent, we emphasize performing a comprehensive evaluation using the three parameters. The OCA is the most accurate parameter, the MCD has the highest sensitivity, and the OCD is a good assistant for the MCD.

**Figure 3:**
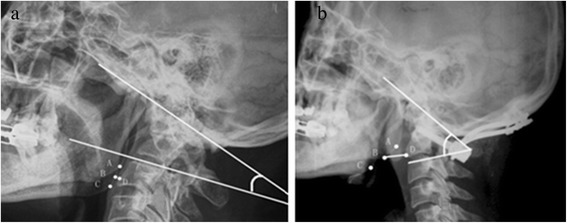
**A preoperative and postoperative radiograph. (a)** Preoperative radiograph of a patient with chronic atlantoaxial dislocation secondary to occipitalization showed a decreased MCD and OCA. After surgery **(b)**, the MCD, OCA, and the curve of the cervical spine were all improved, although the MCD was somewhat longer than normal.

It should be noted that the subjects in our study were people with normal cervical spines, just like those in Phillips’ group. For patients with severe occipitocervical deformity, compensatory morphological changes in the lower cervical spine should be considered together with the occipitocervical position. Figure 3a shows a patient with chronic atlantoaxial dislocation secondary to occipitalization. The small OCA was caused by cervical hyperlordosis and anterior rotation of the head. Although anterior translation of the atlas should increase the MCD, anterior rotation of the head counteracted anterior translation, resulting in a short MCD. After surgery (Figure 3b), we achieved an OCA within the normal range (53°), and the curve of the cervical spine was improved at the same time. The MCD was longer than normal, which might have been caused by incomplete reduction of the atlas and decreased cervical lordosis. The OCD was obviously shortened, mainly because of the poorly developed atlas. Therefore, we concluded that the occipitocervical neutral position varies in severe pathological conditions, and the values offered in this paper should be used conditionally for such cases. Whether the values can be restored to within a normal range depends on correcting the individual patient’s basic pathology. It would be poor practice to pursue normal measurement values without correcting the primary pathology.

Another limitation of this study is that some of the demographic characteristics of the study sample (e.g., body size, weight) were not included due to imperfections in the clinical database. As such, prospective studies are still needed to obtain more reliable measurements.

## Conclusions

This study offers reference values for OCA and OCD for the estimation of the occipitocervical neutral position. The introduction of the MCD made the evaluation more direct and more comprehensive. The long-term results of occipitocervical fusion are expected to be improved when the OCA, OCD, and MCD are adjusted according to the lateral radiograph during surgery. However, considering the complicated anatomic characteristics of the investigated region, it is more justified to say that the method used in this study serves as a proposed approach rather than a universal treatment regimen.

## Abbreviations

BCR: Body/canal ratio
MRI: Magnetic resonance imaging
CT: Computed tomography
OCA: The occipitocervical angle
OCD: The occipitocervical distance
MCD: Mandible cervical distance
